# Pelvic Inflammation and the General Practitioner

**Published:** 1891-09

**Authors:** 


PELVIC INFLAMMATION AND THE GENERAL PRACTITIONER.—The pathology of pelvic inflammation is settled beyond cavil. Non-puerperal pelvic inflammation is peritonitis, non-puerperal pelvic cellulitis does not exist, or exists only as a curiosity—resulting from traumatism and infection of the pelvic connective tissue. These statements are also nearly true of puerperal pelvic inflammation. Puerperal peritonitis is common; puerpereal cellulitis is rare. It is almost equally well known that pelvic peritonitis almost invariably means salpingitis with infection of the peritoneum by discharge of the tubal contents at the fimbriated end of the tube; and that recurring attacks of pelvic peritonitis are due to leaky tubes. Other causes of pelvic peritonitis are changes taking place in pelvic tumors, especially dermoid cysts, traumatism and perhaps the effect of exposure at the menstrual periods. These facts have been known for thirty years, having been discovered by Bernutz, and they have been demonstrated fully by the surgery of to-day.

It is also equally well known that chronic pelvic peritonitis does not tend to a natural cure, and that recurrences of acute peritonitis are characteristic of the disease, being due to leakage from the focus of poison, the diseased tubes. The natural end of such cases is invalidism or death during a recurrent acute peritonitis. Experience has shown the medical treatment, while affording much palliative benefit, is inadequate to effect a cure.

Ablation of the diseased uterine appendage or appendages, the focus of the disease, is the only treatment offering a radical cure. When operation is done early, before the patient is a wreck, the immediate risk to life is slight, less than five per cent., and prompt restoration to health follows. Operations done on such diseased structures does not in any way lessen the fecundity of women; they are sterile by reason of the disease. It restores such sufferers to health and usefulness; saving some from invalidism and others from death. These facts are now amply demonstrated and are opposed only by those in the profession who object to everything that has not the stamp of antiquity upon it.

What, then, becomes the duty to the practitioner when called to attend these cases? To recommend operation by skillful hands. No more pressing duty devolves upon him' and it is time that the matter is brought home to the practitioner. He should know and feel that he is neglecting his duty when he puts such cases to bed, treats the acute attacks by opium internally and poultices externally; and between attacks paints the vagina with iodine and inserts the glycerin tampon. Such neglected cases are brought later to the surgeon either as broken-down wrecks, or as women dying with suppurative peritonitis. These are the "too late" cases. Either their nutrition is so profoundly depraved that they require months to recuperate, or being past recuperation they remain wrecks, or swell the death-list of the surgeon who is too conscientious to refuse them a last chance. Hereafter the responsibility for such maltreatment will rest with the family physician.

The cases in which hesitancy may be felt are cases of slight or catarrhal salpingitis, in which the fimbræ are not agglutinated, and in which accumulation of tubal secretions has not occurred. These cases are subjects of watchful care. Such of them as progress from bad to worse may be referred to the surgeon. The others are fair subjects for medical treatment, so long as life is threatened by attacks of peritonitis—*Med. and Surg. Reporter.—Southern Clinic.*

